# miR-151 Affects Low-Temperature Tolerance of *Penaeus vannamei* by Modulating Autophagy Under Low-Temperature Stress

**DOI:** 10.3389/fcell.2021.595108

**Published:** 2021-04-09

**Authors:** QingJian Liang, WenNa Dong, MuFei Ou, ZhongHua Li, Can Liu, FeiFei Wang, Yuan Liu, WeiNa Wang

**Affiliations:** Guangzhou Key Laboratory of Subtropical Biodiversity and Biomonitoring, Guangdong Provincial Key Laboratory for Healthy and Safe Aquaculture, Key Laboratory of Ecology and Environmental Science in Guangdong Higher Education, College of Life Sciences, South China Normal University, Guangzhou, China

**Keywords:** autophagy, low temperature, *P. vannamei*, miR-151, target of rapamycin

## Abstract

MicroRNAs (miRNAs) play key roles in many physiologic and pathologic processes, including autophagy. Autophagy is cellular in an emergency response mechanism of environment stress, but their complex molecular regulatory mechanism under low-temperature stress is largely unknown in shrimp, especially miRNA-mediated regulation of autophagy in low-temperature tolerance. In this article, a shrimp *PvTOR* and miRNA pva-miR-151 cooperation in response to low-temperature stress has been reported. Pva-miR-151 showed expression patterns opposite to target *PvTOR* under low-temperature stress. The pva-miR-151 targets the 3′-UTR region of *PvTOR*, regulate the formation of autophagosome, which contribute to the degradation and recycling of damaged organelles. In addition, the low-temperature tolerance was correlated positively with autophagy in shrimp. Silenced pva-miR-151 increased sensitivity to low-temperature stress, whereas overexpression pva-miR-151 decreased the expression of PvTOR and p-TOR and increased tolerance to low-temperature stress by improving the formation of autophagosome and total hemocyte count. In addition, the TOR activator 3BDO can partially rescue autophagy induced by overexpression of pva-miR-151; these results indicate that miR-151 was necessary for the low-temperature tolerance in shrimp. Taken together, we provide a novel strategy and mechanism for shrimp breeding to improve shrimp low-temperature tolerance.

## Introduction

Crustaceans have developed a complete protection system during the long-term evolution that responds to external factors by adjusting their vital functions to current needs (Lago-Lestón et al., 2007). There are external factors such as water temperature, salinity, dissolved oxygen, pH, nutrition, and stress (Wang et al., 2009; Qiu et al., 2011; Liang et al., 2019b). Low temperature is one of the main stress factors for aquaculture. *Penaeus vannamei* is one of the most important cultured shrimp in the world and has been adversely affected by low temperatures. Frequent periods of low temperatures have led to large-scale economic losses for several decades (Zhou et al., 2011; Li et al., 2013). Recent studies have shown that low-temperature stress directly affects the survival, growth, and metabolism and also induces the generation of reactive oxygen species (ROS), hemocyte apoptosis, and autophagy, reduces the immune functions, and causes DNA damage in shrimp (Cheng et al., 2005; Qiu et al., 2011; Li et al., 2013). Thus, studies on cold-adaptation mechanisms to enhance its cold tolerance will be great value to the aquaculture industry of *P. vannamei*.

Many stresses could trigger autophagy, and autophagy becomes a strategy to adapt and copes with stress (Marino et al., 2014). It is an essential process for eukaryotes, which via the lysosome degrade cytoplasm and organelles to recycle misfolded proteins or damaged organelles to resynthesize macromolecules and/or ATP generation (Wang and Klionsky, 2003; Tettamanti et al., 2008). Therefore, autophagy is a self-survival mechanism under external stress, hypoxia, and endoplasmic reticulum stress. Previous studies have shown that hypothermia can cause an increase in the number of liver lysosomes and autophagosomes, endoplasmic reticulum disorder, and swelling of mitochondria (Salas et al., 1977). Fish oil alleviated liver injury induced by intestinal ischemia/reperfusion via AMPK/SIRT-1 autophagy pathway (Jing et al., 2018). In addition, autophagy plays a vital role in the innate immunity of *P. vannamei* (Wu et al., 2019). The mechanistic target of rapamycin (mTOR), an atypical serine/threonine protein kinase that belongs to the phosphatidylinositol-3-kinase (PI3K)–related kinase (PIKK) family, is a key molecule in the process of autophagy induction (Hu et al., 2015). There is increasing evidence that mTOR signaling pathway is inactivated in response to diverse environmental (Laplante and Sabatini, 2012; Sun et al., 2015). Subsequently, the inactivated TOR causes the activation of ULK1 to initiate phagophore formation, and the beclin1–Vps34 complex extends the nascent autophagosome to form a mature double-membrane autophagosome. Finally, autophagosomes fuse with lysosomes to degrade cytoplasmic content, thereby recovering energy for cells (Mukhopadhyay et al., 2020). In addition, researchers found that mTOR in the balance between growth and autophagy is crucial when organisms respond to environmental stress (Liu et al., 2018b). However, the underlying regulatory mechanism of TOR/autophagy pathway in low-temperature stress that induced hepatopancreas injury remains incompletely understood.

MicroRNAs (miRNAs) are a family of small non-coding RNA about 20–24 nt and inhibit the expression of their target mRNA at posttranscriptional level by directly binding to 3′-UTRs (Carthew and Sontheimer, 2009). MiRNA has been reported to participate in the various biological and metabolic processes in cells, such as development, metabolism, apoptosis, autophagy, and signal transduction, which are associated with abiotic and/or biotic stress responses (Plasterk, 2006; Zeng et al., 2015). Recent studies suggest that 63 miRNAs of shrimp responding to WSSV infection have been identified (Huang and Zhang, 2012). Twenty-four miRNAs of shrimp were closely related to phagocytosis, apoptosis, and the pro-phenoloxidase system (Yang et al., 2012). Studies have found that increased susceptibility to arrhythmias in response to acute myocardial estrogen deprivation is dependent on the up-regulation of miR-151 in the rat (Zhang et al., 2013), and miR-151-3p is closely related to improved survival in resected cholangiocarcinoma (McNally et al., 2013), suggesting that miR-151 is a vital factor. In addition, STAT is a typical transduction factor involved in the regulation of autophagy. MiR-151 could target 3′-untranslated region (3′-UTR) of STAT3 gene, which suggests a possible connection between miR-151 and autophagy regulation (Liu et al., 2018a). However, little is known about the molecular mechanism of miR-151 and autophagy.

In our study, RNA-seq was used to screen the miRNAs involved in autophagy to respond to low-temperature stress. The present study showed that in shrimp miRNAs, which were conserved in animals, the level of miR-151 increased significantly in low-temperature stress. Application of abnormal expression, transmission electron microscope (TEM), cumulative mortality, etc., connect shrimp autophagy with the low-temperature challenge. Finally, using a luciferase reporter assay identified miR-151 directly targeted mTOR to regulate autophagy. This study provided a novel insight that miRNA could activate autophagy to alleviate hepatopancreas injury induced by acute low-temperature stress in *P. vannamei*.

## Materials and Methods

### Shrimp Culture and Low-Temperature Stress

Healthy *P. vannamei* were cultured in 200 L (approximately 3‰ salinity) aquariums at 23°C–25°C, pH 7.4, continuous aeration, which with length range 6.1 ± 0.6 cm and weight 5.2 ± 0.9 g, were obtained from a shrimp farm in Panyu, Guangdong Province, China. All animal procedures were licensed under the Institutional Animal Care and Use Committee of the Institute of Zoology, Chinese Academy of Sciences.

From healthy shrimp that had been acclimated to 25°C ± 0.5°C for 2 weeks, 30 shrimp were randomly assigned in different groups and were directly transferred to 12°C ± 0.5°C for acute low-temperature stress, 25°C ± 0.5°C as the control group. At different times after low-temperature stress (0, 1.5, 3, 6, 12, and 24 h), there were randomly collected three shrimp tissue samples for each group. Different tissue samples were immediately frozen in liquid nitrogen and stored at -80°C.

### Quantitative Real-Time Polymerase Chain Reaction (RT-qPCR)

Total RNA was extracted using Trizol (Invitrogen) according to the manufacturer’s recommendations and previous description (Liang et al., 2019c). The concentration and quality of the RNA were determined by the 260/280-nm spectrophotometer (NanoDrop Technologies, Montchanin, DE) and 1.5% agarose gel electrophoresis. First-strand cDNA was synthesized from total RNA (1 μg in each reaction) using a PrimeScript RT reagent kit with gDNA Eraser (Takara, Dalian, China) according to the manufacturer’s protocol. Quantitative polymerase chain reaction (qPCR) used an ABI 7500 thermal cycler (Applied Biosystems, Foster City, CA) using SYBR Premix Ex Taq (Takara) in a 20-μL reaction volume with three biological and three technical replicates. Shrimp small nuclear RNA (U6), eukaryotic elongation factor 1α, and β-actin were used as endogenous controls. All gene-specific primers used in this study were designed using Primer Premier 5.0 (Supplementary Table 1).

### miRNA Library Analysis

The RNA was enriched in a size range of 18–30 nt by polyacrylamide gel electrophoresis (PAGE). Then, the 3′ adapters were added, and 5′ adapter RNAs were sequenced using Illumina HiSeq^TM^ 2500 by Gene *De novo* Biotechnology Co. (Guangzhou, China). The data from the miRNA-seq are available on NCBI (NO. PRJNA560613). Clear data normalization was performed with the weighted regression method. False discovery rate (FDR) ≤ 0.001 and | log_2_FC| ≥ 2.5 miRNA were chosen for further analyses. The heat map of differentially expressed miRNAs expression profile was drawn with ggplots heatmap 0.2. The RNAhybrid v2.1.2^1^, TargetScan 7.0^2^, and miRanda 3.3^3^ were used to predict targets of miRNAs with default parameters. Enrichment analysis of the predicted target genes was conducted with Gene Ontology (GO)^4^ and Kyoto Encyclopedia of Genes and Genomes (KEGG) pathway^5^.

### Gene Cloning and Sequence Analyses

The primary transcript of pva-miR-151 (pri–pva-miR-151) and 3′-UTR were isolated from the shrimp cDNA library by PCR using PrimeSTAR^®^ Max DNA polymerase (Takara). The PCR product was verified by Sanger sequencing. The mfold Web server^6^ was used to detect hairpin structures with pre-miRNA sequence. By using the ClustalW2 program, multiple sequence alignment can be performed. On the basis of the sequence alignment and using the neighbor-joining algorithm as implemented in the MEGA 5.1 software package, a phylogenetic tree was constructed, with 1,000 bootstrap replications.

### Silencing or Overexpression of miR-151 in Shrimp

To silence miR-151, the antago-151 (5′- CCUCAAAGAGCUU CAGUCCAGU-3′) and antago normal control (NC) (5′- CAGUACUUUGUGU AGUACAA-3′) were synthesized (GenePharm, Shanghai, China) with a 5′ and 3′ phosphorothioate backbone, a 3′ end cholesterol modified, and a 2′-O-methyl modification at all nucleotides. The antago-151 (2 nM/g) and a control antago NC were injected intramuscularly into healthy shrimp. After low-temperature stress, the tissue of three randomly selected shrimp was collected at different time points.

For overexpression of miR-151, ago-151 (5′- ACUGGACU GAAGCUCUUUGAGG-3′) and ago NC (5′- UUCUCCGA ACGUGUCACGUTT-3′) were synthesized *in vitro*. The formation of double-stranded RNAs was modified with a 5′ and 3′ phosphorothioate backbone, a 3′ end cholesterol modified, and a 2′-O-methyl modification at the anticoding strand nucleotide. The ago-151 (2 nM/g) and a control ago NC were injected intramuscularly into healthy shrimp. After low-temperature stress, the tissue of three randomly selected shrimp was collected at different time points. All the assays were biologically repeated three times.

### Total Hemocyte Count

Hemolymph samples were analyzed by using a hemocytometer. A drop of diluted hemolymph sample with anticoagulant (4.2 g ⋅ L^–1^ sodium chloride, 8 g ⋅ L^–1^ sodium citrate, 20.5 g ⋅ L^–1^ glucose, pH 7.5) was placed in a hemocytometer and observed under a light microscope (Olympus CX31RBSF, Beijing, China) to measure total hemocyte count (THC). Each sample was quantified nine consecutive times. THC was calculated as cells per milliliter.

### Primary Hemocyte Culture

Primary hemocyte culture was made following a published protocol with minor modification (George et al., 2011; Thansa et al., 2016). Hemolymph was withdrawn into a syringe half-filled with Alsever solution as anticoagulant and then centrifuged at 2,500 rpm for 5 min at 4°C. The cell pellet was roughly washed with 10% sodium acetate twice before being washed once with culture medium [2 × Leibovitz’s L-15, 100 units/mL penicillin, 100 mg/mL streptomycin, 10% each of fetal bovine serum (FBS), and shrimp meat extract]. In order to prepare shrimp muscle extract, 10 g muscle tissue was dissected from surface sterilized live shrimp and homogenized in 100 mL with phosphate-buffered saline (PBS), and centrifuged at 4,500 × *g* for 5 min to remove cell debris. The supernatant was again centrifuged at 4,500 × *g* for 10 min to remove the coagulated proteins, sterilized by sequentially passing through filters (0.45 followed by 0.22 μm), and kept at 4°C until being mixed with the cell culture medium. Then, a culture medium was used to resuspend the pellet, and cells were seeded at the density of 5 × 10^6^ cells/well in a 24-well plate. Trypan blue staining was used to determine the cell viability of primary hemolymph. If the positive result of trypan blue staining of primary hemolymph was less than 5%, cells isolated from that shrimp would be further used in the experiments.

### Dual-Luciferase Reporter Assay

The 3′-UTR fragments were cloned into psiCHECK^TM^-2 vector (Promega, United States) as a Renilla luciferase reporter vector, which contains the predicted pva-miR-151 target sites of *PvTOR*, using the *Xho*I and *Not*I site with primers in Supplementary Table 1. To further identify the binding site of pva-miR-151, a mutated site was also constructed into psiCHECK^TM^-2 vector by ClonExpress^®^ Ultra One Step Cloning Kit (Vazyme, Nanjing, China). The overexpression of pva-miR-151 or pva-pre-miR-151 used pcDNA^TM^ 6.2-GW/miR vector (Invitrogen, Carlsbad, CA, United States) in hemocyte. Using FuGENE^®^ HD Transfection Reagent (Invitrogen) according to the manufacturer’s instructions, cotransfection was done with luciferase reporter vector and pcDNA-miR-151, or pcDNA-pre-miR-151, respectively. After transfection at 48 h, activities of the luciferases were measured using the Clarity^TM^ Western ECL substrate (Bio-Rad, United States) with EnSpire^TM^ multiscan spectrum (PerkinElmer, Germany). Data were represented as the ratio of Renilla to firefly luciferase activity with three independent replicates after normalization.

### Flow Cytometer Detected Hemolymph Autophagy Flux

Hemocyte autophagy fluxes were quantified using the commercial Cyto ID^®^ autophagy detection kit (ENZO Life Sciences, ENZ-KIT175-0200) following the manufacturer’s protocol. Briefly, the primary hemocytes (1 × 10^6^ cells) were incubated for 24 h with dimethyl sulfoxide (DMSO), 2 μM rapamycin (Rap), 50 μM 3BDO, 8 μM chloroquine (CLQ), and 0.4 μg empty vector or pcDNA-miR-151 according to specific experiments. The cells were washed with PBS, and then suspended cells were resuspended using 1 mL buffer containing 5% FBS and Cyto ID^®^ Green Detection Reagent. Incubation was performed for 30 min under room temperature (25°C). Cells were washed with DPBS once, and cells were resuspended in PBS containing 2% FBS. Cyto ID^®^ fluorescence was detected using BD FACSAria III flow cytometer (BD Biosciences); data of cell counts were plotted as fluorescein isothiocyanate (FL1) fluorescence intensity. The experiment was performed in triplicate.

### Histological Analysis by Hematoxylin–Eosin Stain

For all experiments, at least three samples were stained per treatment sampled. Hepatopancreas was fixed with the 4% paraformaldehyde fixative for 24 h and subsequently replaced by ethanol for at least 24 h using the methods described (Ng et al., 2018). The hepatopancreas was then sectioned and stained with hematoxylin–eosin stain (H&E) following standard histological methods. Hepatopancreas structures were observed using light microscopy (DM6, Germany).

### Western Blotting

Hepatopancreas was lysed in lysis RIPA buffer (Beyotime, China) for 30 min on ice. After incubation, lysates were centrifuged (14,000 × *g*, 15 min) at 4°C, and each supernatant was collected. Total protein was quantified using the traditional BCA method. Fifty micrograms of protein was separated by 15% sodium dodecyl sulfate–PAGE and electrotransferred onto polyvinylidene difluoride membrane (Bio-Rad). The membranes were incubated in 20 mM tris-buffered saline (TBS) containing 5% BSA for 1 h at room temperature. The membranes were washed with tris-buffered saline and tween 20 (TBST) for 5 min and incubated with the anti-LC3I/II (1:500, Abcam), beclin1 (1:1,000, CST), TOR (1:500, CST), p-TOR (1:500, CST), and actin (1:1,000, CST) at 4°C overnight in a shaking incubator and then washed with (3 × 5 min per wash), and the protein was detected using alkaline phosphatase (1:2,000) as the secondary antibody was incubated for 2 h at room temperature. The membrane was detected with 5-bromo-4-chloro-3-indolyl-phosphate/nitroblue tetrazolium substrate (Sangon Biotech, Shanghai, China). To quantify the signals, gray intensities of Western blots were calculated using ImageJ^7^.

### Transmission Electron Microscopy

Transmission electron microscopy (TEM) was used to observe the cell ultrastructure. Changes regarding mitochondrial state and nuclear condensation indicated cellular damage, and the formation of autophagosome indicated autophagy. Tissues were first fixed with 2.5% glutaraldehyde and subsequently fixed with 1% osmium tetroxide. Cells were embedded in Epon after dehydration with different concentrations of alcohol. Ultrathin sections (0.5 μm) were generated for observation under a transmission electron microscope (JEOL, United States) at 100 kV.

### Statistical Analyses

All data analyses were used SPSS (version 20.0 for Windows). The data were expressed in the form of mean ± SD. Different groups of data were analyzed by using *t* test. We set the threshold for statistical significance at *p* < 0.05.

## Results

### miRNAs Involved in Low-Temperature Response of Shrimp Hepatopancreas

**FIGURE 1:**
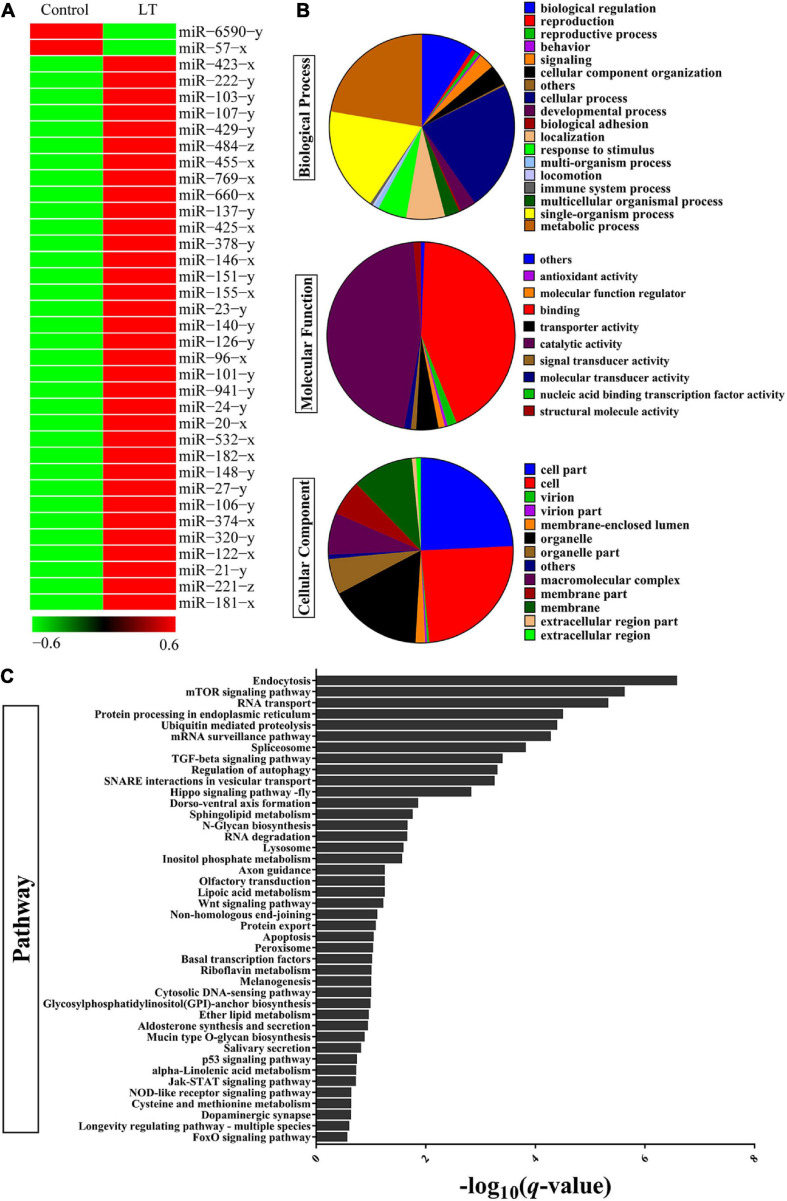
Identification of differentially expressed miRNAs and target gene in shrimp response to low-temperature stress. **(A)** Heat map analysis of significant DE miRNAs after low-temperature stress. Low-temperature stress 3 h later, the miRNAs of shrimp hepatopancreas were subjected to miRNA library analysis. **(B)** GO and **(C)** KEGG pathway analysis of significant DE miRNAs target gene after low-temperature stress.

To evaluate the miRNAs associated with the low-temperature response, the expression profile analysis was performed on miRNA or mRNA extracted from the hepatopancreas of adult shrimp, which were subjected to low-temperature stress for 3 h. Hundreds of shrimp miRNAs were identified by the hepatopancreas expression profiles. MiRNAs with a threshold of the FDR ≤ 0.001 and an absolute value of | log_2_FC| ≥ 2.5 were chosen for further analyses. The results indicated a dynamic regulation of 36 miRNAs (*P* < 0.05) during the period in which there was low-temperature stress at 3 h (Figure 1A and Supplementary Table 2). To further understand the function of miRNA through the predicted target genes (Supplementary Table 3), GO and KEGG pathway enrichment analysis was performed. In terms of biological process class, it was found that the response to a stimulus may be associated with the antistress capability. In the field of molecular function, approximately half of the genes were involved in catalytic activity, and antioxidant activity was found (i.e., oxidoreductase, peroxidase) (Figure 1B). In terms of pathway classification, both endocytosis and an mTOR signaling pathway were significantly enriched (Figure 1C and Supplementary Table 4). Finally, the comprehensive analysis found that the most significant of miRNA in combination with the mTOR 3′-UTR was miR-151. Thus, miR-151 was selected, which may be involved in TOR signal pathway in response to stress for further analyses.

### Characteristics of miR-151

**FIGURE 2:**
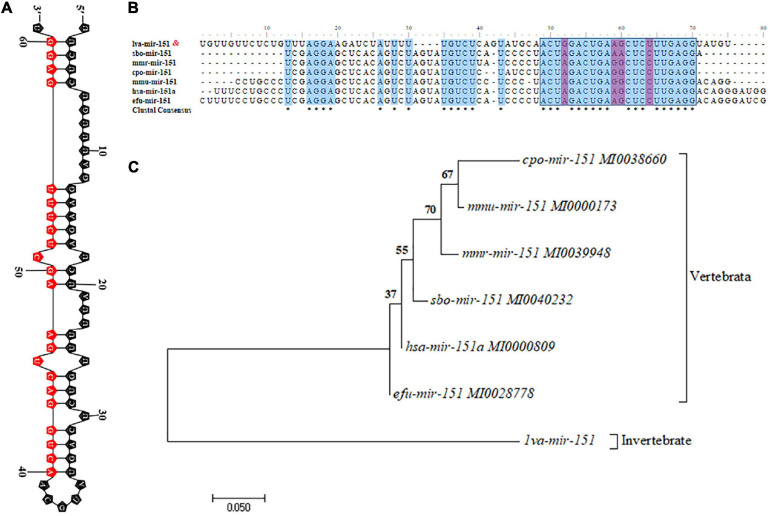
Characteristic analysis of miR-151. **(A)** The hairpin structures of pre-miR-151 from *Epinephelus coioides* and mature miR-151 sequences are indicated in the red box. **(B)** Precursor miR-151 multiple sequence alignment. **(C)** The phylogenetic tree of precursor miR-151 with other species reconstructed by the neighbor-joining (NJ) method using the MEGA 7. The accession numbers used in this analysis are listed in the figure.

The primary transcript of pri–pva-miR-151 was cloned from the hepatopancreas of *P. vannamei*. The pri–pva-miR-151 was composed of 311-nt, which contained a 63-nt precursor sequence (pre–pva-miR-151) and 22-nt mature sequence (pva-miR-151) (Supplementary Table 5). In addition, the secondary structure analysis performed by the mfold Web server predicted a well-developed hairpin structure in the pre–pva-miR-151 sequence (Figure 2A and Supplementary Figure 1). Mature miR-151 and miR-151 precursor were found to be conserved across multiple species (Figure 2B). However, the bases located at positions 4 and 14 of the mature miR-151 sequence are “G” and “U” in mammals. The pre-miR-151 sequences were used to construct a phylogenetic tree, which revealed that miR-151 was divided into two distinct branches invertebrate and vertebrata (Figure 2C).

### Expression Analysis of *PvTOR* and pva-miR-151

**FIGURE 3:**
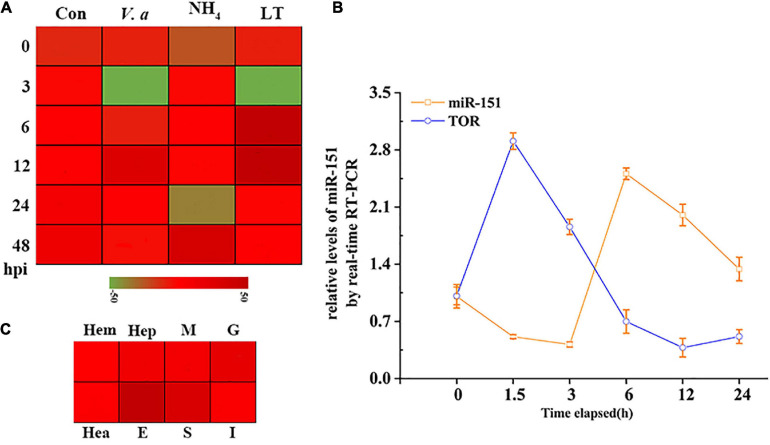
Expression pattern analysis of *PvTOR* and pva-miR-151. **(A)** Transcriptional profiling of pva-miR-151. The values calculated by qRNA data were shown as a heat map. **(B)** Different tissues expression profiles of pva-miR-151 in shrimp. The colors of the bar shown to the right of the heat map varied from red to green, representing the relative expression levels from high to low. Hepatopancreatic (Hep), hemolymph (Hem), heart (Hea), muscle (M), gill (G), stomach (S), intestine (I), and eye (E). **(C)** pva-miR-151 and *PvTOR* expression patterns in shrimp under low-temperature stress. cDNAs were synthesized from total RNA extracted from various tissues of shrimp. The expression levels of pva-miR-151 were normalized using U6, and *PvTOR* was normalized using β-actin. Error bars represented standard deviations of the mean values from three independent experiments.

To further analyze the expression patterns of *PvTO*R and pva-miR-151 in shrimp, the following experiments were carried out in this study. The results showed that the expression of pva-miR-151 was induced by various abiotic and biotic stresses, including ammonia nitrogen, low temperature, and *Vibrio alginolyticus* challenge (Figure 3A). To verify whether these miRNAs and TOR were indeed changed in low-temperature stress, qPCR was used to quantify the levels of these pva-miR-151 and *PvTOR* after low-temperature challenge. The results showed that the expression of pva-miR-151 was decreased at 1.5 h and then increased at 3 to 24 h. An opposite pattern was observed in the expression of *PvTOR* in response to low-temperature stress (Figure 3B). These results indicated that *PvTOR* might be regulated by pva-miR-151 *in vivo* in response to low-temperature stress. In addition, tissue-specific expression of pva-miR-151 showed that pva-miR-151 was expressed in almost all examined tissue types, such as hepatopancreatic, hemolymph, heart, muscle, gill, stomach, intestine, and eye (Figure 3C).

### Low-Temperature Stress Induced Autophagy *in vivo*

**FIGURE 4:**
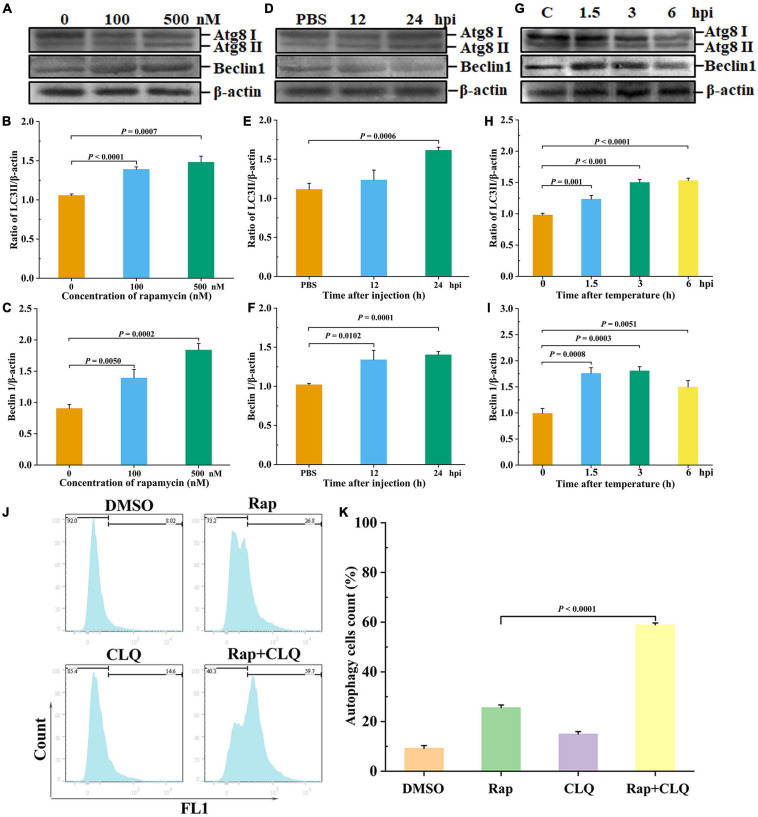
Low temperature induced autophagy *in vivo*. Shrimp were injected with rapamycin at different concentrations. At 12 and 24 h after rapamycin injection, the shrimp hepatopancreas specimens was collected for Western blot analysis with an LC3 and beclin1 antibody. Dosage of rapamycin for autophagy induction Western blot analysis **(A)**, LC3 **(B)**, and beclin1 **(C)** statistical analysis. Time-course detections of autophagy Western blot analysis **(D)**, LC3 **(E)**, and beclin1 **(F)** statistical analysis. Low-temperature stress induction for autophagy Western blot analysis **(G)**, LC3 **(H)**, and beclin1 **(I)** statistical analysis. The ratios of LC3II/β-actin and beclin1/β-actin gray-scale value were calculated with three independent experiments. Shrimp primary hemolymph cells were treated with DMSO, 2 μM rapamycin (Rap), 8 μM chloroquine (CLQ), or Rap + CLQ for 24 h to induce autophagy and then used CYTO-ID^®^ Green Detection Reagent 2 stain for 30 min and analyzed by flow cytometry **(J)** and statistical analysis **(K)**. Data were shown as fold changes and mean ± SD.

LC3 is a key autophagy-related protein, and previous studies showed that LC3 level is closely related to the autophagic flux (Zhu et al., 2017). To evaluate whether autophagy occurred in shrimp, rapamycin (autophagy activator) was used to induce autophagy of shrimp hepatopancreas, followed by the detection of LC3II/β-actin and beclin1. After different concentrations of rapamycin were injected into shrimp, there is a significant increase in LC3II/β-actin ratio, and beclin1 expressions were found in the 100 or 500 nM rapamycin compared to the control group (Figures 4A–C). Nevertheless, high concentration of rapamycin was cytotoxic to shrimp. Therefore, 100 nM rapamycin was chosen for further study. The results showed that autophagy was induced at 24 h after injection of rapamycin compared with the control (Figures 4D–F). These results indicated that autophagy of shrimp hepatopancreas could be activated at an early stage of rapamycin challenge. To explore the effect of low temperature–induced autophagy, the shrimp were treated with low-temperature stress. Western blotting revealed that the autophagy-related proteins LC3 and beclin1 of shrimp hepatopancreas dramatically increased in response to low-temperature stress. At an early stage of low-temperature stress (1.5 h), the shrimp autophagy was induced (Figures 4G–I). The results indicated that low-temperature stress was an inducer of autophagy in shrimp *in vivo*, which was similar to that of rapamycin.

Furthermore, to further determine the occurrence of autophagy, we performed a degradation experiment. Primary cells were treated with mTOR inhibitor rapamycin and lysosomal protease inhibitor chloride. Primary cells were treated with 2 μM rapamycin and 8 μM CLQ for 24 h. CYTO-ID^®^ Green Detection Reagent 2 was stained for 30 min and analyzed by flow cytometry. Compared with untreated cells, the fluorescence was increased, with treatment with RAP + CLQ (Figures 4J,K). The results indicated that autophagy can be induced under stress conditions.

### Alters of pva-miR-151 Impact Low-Temperature Tolerance of Shrimp

**FIGURE 5:**
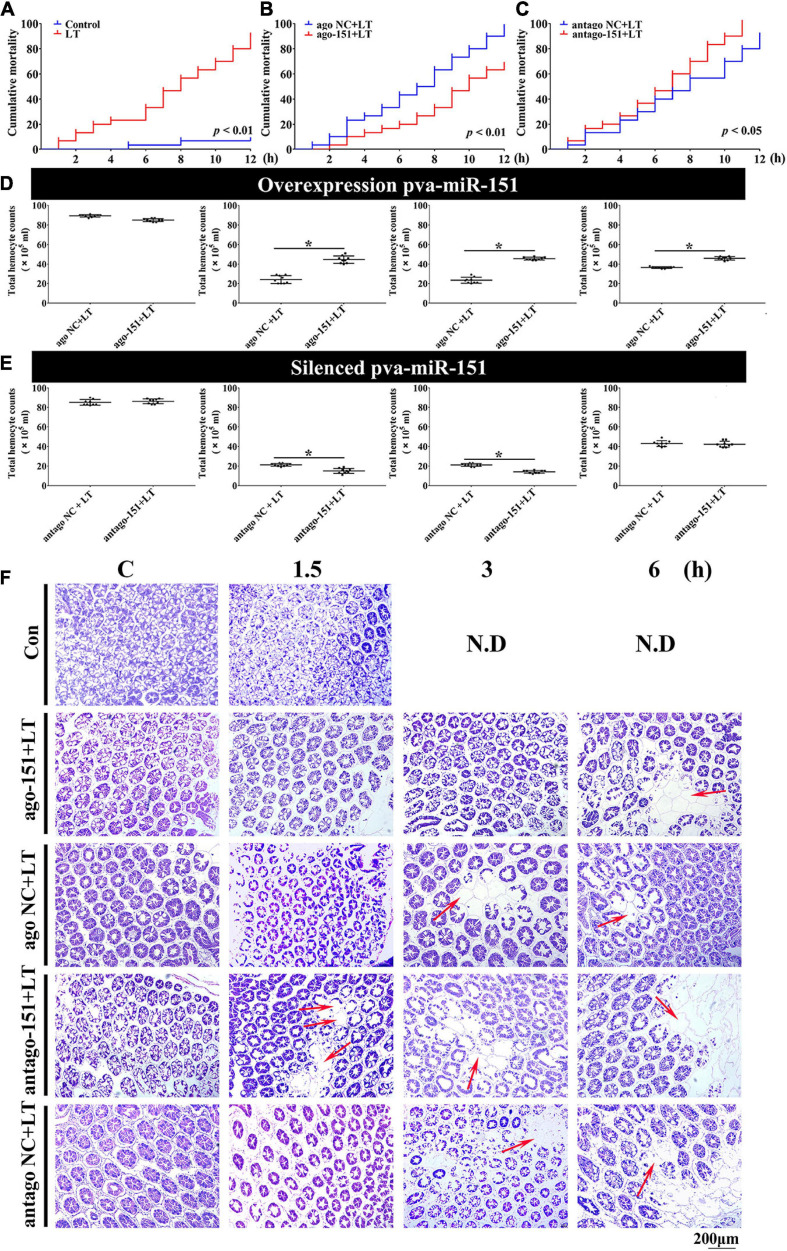
Alterations of pva-miR-151 impact low-temperature tolerance of shrimp. **(A)** The shrimp were treated with low temperature at various times after challenge; the shrimp mortality was examined. In low temperature–challenged shrimp, mortality was reduced after pretreatment with ago-151 **(B)** and increased after pretreatment with antago-151 **(C)**. Differences in mortality curves between each group were analyzed by the log–rank test. THC was increased after pretreatment with ago-151 **(D)** and reduced after pretreatment with antago-151 **(E)**. Differences in THC between each group were analyzed by *t* test. *Significantly different (*p* < 0.05). **(F)** H&E-stained hepatopancreas collected from ago-151, ago NC, antago-151, and antago NC groups after low-temperature challenge at 0–6 hpi. Pretreatment with ago-151 alleviates the vacuole cells, and cell atrophy occurred. ND, no data. Scale bar, 200 μm.

To investigate a possible role of pva-miR-151 in response to low-temperature stress in shrimp, ago-151 or antago-151 was used to overexpress or silence pva-miR-151 in *P. vannamei*, respectively. The efficiency was analyzed by qPCR. We chose the period 24–36 h for the experiment in which the overexpression efficiency was more than 100-fold or the silencing efficiency was less than 50% (Supplementary Figure 2). The results showed that low-temperature challenge significantly increased the mortality of shrimp (*P* < 0.01; Figure 5A). Importantly, overexpression of pva-miR-151 significantly reduced mortality (*P* < 0.01; Figure 5B), while silencing pva-miR-151 significantly enhanced the mortality of shrimp in low-temperature challenge (*P* < 0.05; Figure 5C).

Previous studies have shown that loss of circulating hemocytes or hemocyte DNA damage would endanger survival (Li et al., 2013; Liang et al., 2019a). Thus, this possibility was observed, and results showed that overexpression of pva-miR-151 significantly elevated THC, while silencing pva-miR-151 significantly reduced the THC in low-temperature challenge (Figures 5D,E). In addition, the hepatopancreas is a sensitive organ that can show ultrastructural alterations at the early stages of stress (Xu et al., 2018). Low temperature caused the vacuoles cells to increase and cell atrophy. Excitingly, silencing pva-miR-151 appeared to accelerate the lesions such that the vacuole cells occurred at 1.5 h, while vacuoles cells have occurred in 3 h in the NC group. Conversely, in overexpression of pva-miR-151, vacuole cells were the first to appear, at 6 h, after temperature challenge compared with the NC group (Figure 5F and Supplementary Figure 3). All these data indicated that pva-miR-151 protects against damage in response to low-temperature stress in shrimp.

### Pva-miR-151 Affects Low-Temperature Tolerance of Shrimp by Inducing Autophagy

**FIGURE 6:**
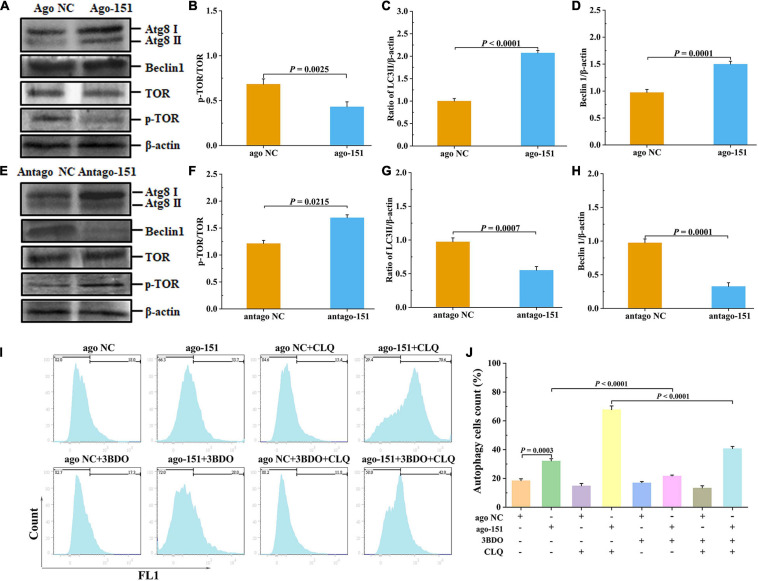
Pva-miR-151 affects low-temperature tolerance of shrimp by inducing the occurrence of autophagy. Shrimp were injected with 2 nM/g ago-151 or antago-151. After 24 h, the shrimp hepatopancreas was collected for Western blot. The Western blot evaluation of shrimp autophagy after ago-151 treatment **(A)**, p-TOR **(B)**, LC3 **(C)**, and beclin1 **(D)** statistical analysis. The evaluation Western blot of shrimp autophagy after antago-151 treatment **(E)**, p-TOR **(F)**, LC3 **(G)**, and beclin1 **(H)** statistical analysis. β-Actin was used as the control. The ratios of pTOR/TOR, LC3II/β-actin, and beclin1/β-actin gray-scale value were calculated with three independent experiments. Shrimp primary hemolymph cells were treatments with DMSO, 20 pmol NC or miR-151, 8 μM chloroquine (CLQ), and 50 μM 3BDO for 24 h to induce autophagy and then used CYTO-ID^®^ Green Detection Reagent 2 stain for 30 min and analyzed by flow cytometry **(I)** and statistical analysis **(J)**. Data were shown as fold changes and mean ± SD.

To further investigate the biological role of pva-miR-151 under low-temperature stress, ago or antago was used to overexpress or silence pva-miR-151 in shrimp. The results showed that overexpression of pva-miR-151 inhibited the protein levels of p-TOR (Figures 6A,B). Importantly, silenced pva-miR-151 reversed the inhibitory effect on TOR signaling pathway (Figures 6E,F). Based on the above results and those from previous studies, the hypothesis presented that low-temperature stress induced the occurrence of autophagy and increased the expression of pva-miR-151 to decrease the protein level of p-TOR. With the decrease of PvTOR activation, the accumulation of autophagy increases, thereby alleviating the damage of the hepatopancreas under low-temperature stress. To confirm our hypothesis, the LC3I/II and beclin1 protein levels were determined in aberration shrimp. The results disclosed that overexpression of pva-miR-151 induced expression of LC3II/β-actin and beclin1 protein level when shrimp were exposed to low-temperature stress (Figures 6A,C,D). On the contrary, silenced pva-miR-151 had an opposite result (Figures 6E,G,H).

To further determine the role of the miR-151 in low-temperature stress–induced autophagy, we performed a degradation experiment. Primary cells were treated with mTOR inhibitor rapamycin and lysosomal protease inhibitor chloride. Primary cells were treated with 50 μM 3BDO, 8 μM CLQ, ago NC and ago-151, for 24 h. CYTO-ID^®^ Green Detection Reagent 2 was stained for 30 min and analyzed by flow cytometry. Overexpression miR-151 in primary cells and treating them with or without 3BDO were used to detect the autophagy flux. The autophagy flux was significantly increased by overexpression miR-151, but suppressed with 3BDO and miR-151 coprocessing treatment cells (Figures 6I,J). Excitingly, the autophagy flux was significantly increased by overexpression miR-151 and CLQ coprocessing, but significantly suppressed with 3BDO, miR-151, and CLQ coprocessing treatment cells (Figures 6I,J).

**FIGURE 7:**
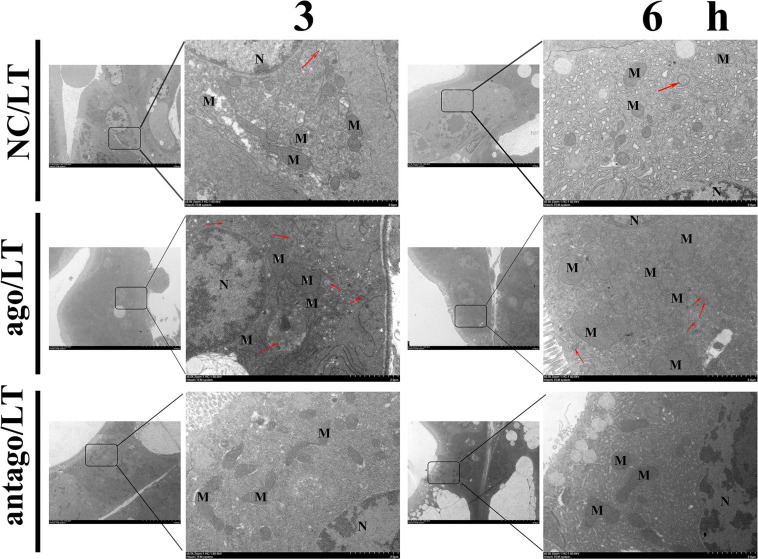
TEM observation the hepatopancreas ultrastructure after pretreatment with ago-151 or antago-151. Mitochondria (M) are round or elongated, the matrix electron density is higher, the ridges are aligned, and there is obvious autophagy (red arrow) after pretreatment with ago-151. Nuclear (N) shrinkage and mitochondria (M) are sparsely distributed, with varying oval sizes, and the ridges are slightly expanded, disordered, and broken after pretreatment with antago-151.

In addition, ultrastructural observation of the hepatopancreas by TEM showed that the overexpression pva-miR-151 induced autophagy formation when shrimp were exposed to low-temperature stress, reducing damaged organelle fragments. For example, mitochondria are round or elongated, the matrix electron density is higher, and the ridges are aligned after pretreatment with ago-151. On the contrary, silenced pva-miR-151 had an opposite result (Figure 7). Nuclear (N) shrinkage and mitochondria are sparsely distributed, with varying oval sizes, and the ridges are slightly expanded, disordered, and broken after pretreatment with antago-151.

All these data indicated that when shrimp were exposed to low-temperature stress, the up-regulated expression of pva-miR-151 inhibited PvTOR, leading to a decrease in p-TOR expression level and the elevation of autophagy formation, which further resulted in increased scavenging of the damaged organelle injury accumulation by low-temperature stress induced in shrimp hepatopancreas cells.

### *PvTOR* Is an Immediate Target of pva-miR-151

**FIGURE 8:**
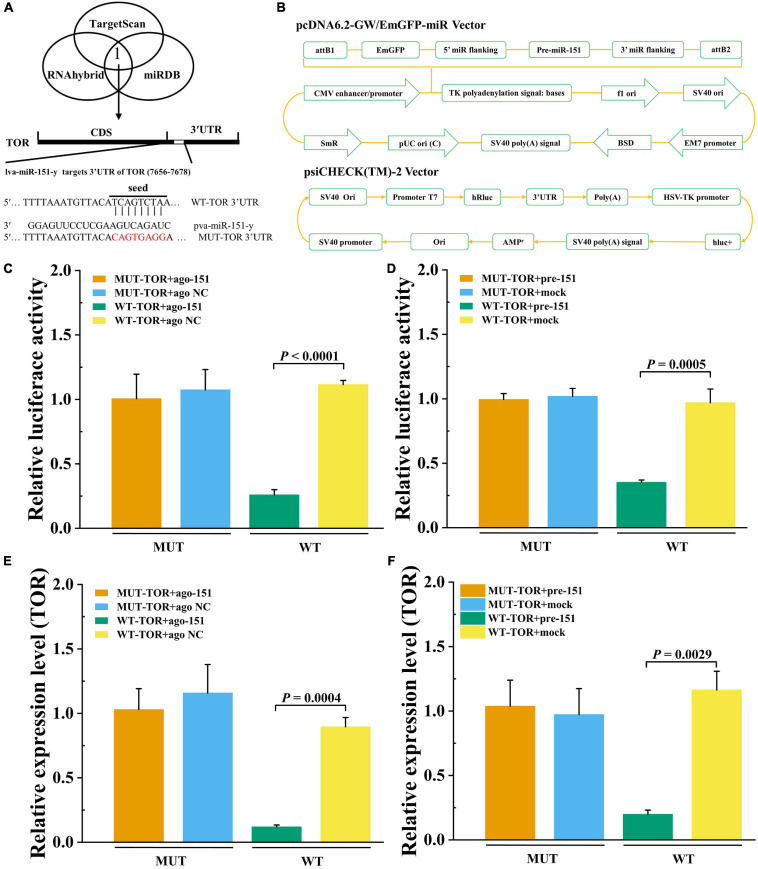
Prediction and validation of pva-iR-151 target genes *PvTOR*. **(A)** Predicted target genes of pva-miR-151. The 3′-UTR of the PvTOR gene was predicted to be targeted by miR-13b used TargetScan, miRDB, and RNAhybrid. Schematic diagram miR-151 to binding 3′-UTR of PvTOR and wild or mutant type 3′-UTR of PvTOR. **(B)** Schematic diagram of pcDNA-pre-miR-151 and psiCHECK-3′-UTR_PvTOR_ vector. The luciferase activity was determined using reporter gene assay. Data were shown as fold changes and mean ± SD. Primary cells were transfected with ago-151 **(C)** or pcDNA-pre-miR-151 **(D)** and psiCHECK-3′-UTR_PvTOR_ cotransfection with Lipofectamine 2000. After incubation at 27°C for 24 h, the luciferase activity was determined. The mTOR expression levels after primary cells were transfected with ago-151 **(E)** or pcDNA-pre-miR-151 **(F)**. Data were shown as fold changes and mean ± SD.

To detect that *PvTOR* was the target gene of pva-miR-151, using the bioinformatics software TargetScan, RNAhybrid, and miRDB intersection was carried out to predict results. The predicted binding site between miR-151 and *PvTOR* 3′-UTR was shown at 7656–7678 (Figure 8A). The pre-miR-151 was cloned into a pcDNA6.2 GW miR vector, and *PvTOR* 3′-UTR was cloned into a luciferase reporter plasmid (Figure 8B). The luciferase reporter assay results showed that overexpression of miR-151 significantly suppressed luciferase activity of reporter genes containing 3′-UTR-WT of *PvTOR* (Figure 8C), and the transfection of pre-miR-151 had similar results (Figure 8D). In addition, overexpression miR-151 significantly suppressed the mRNA expression of *PvTOR* (Figure 8E), and the transfection of pre-miR-151 had similar results (Figure 8F). These results suggested that miR-151 bound directly to the predicted binding site(s) in the *PvTOR* 3′-UTR and negatively regulated *PvTOR* expression.

## Discussion

In nature, aquatic organisms trigger adjustments to their physiology and behavior to adapt to the fluctuation environment (de Souza et al., 2016). Therefore, temperature is one of the most important environmental factors for aquatic animals. Crustaceans are cold-blooded thermophilic animals whose body temperature changes with the water temperature of living waters. As a sensitive organ, ultrastructural alterations can occur in the hepatopancreas at the early stages of stress (Xu et al., 2018). Recent studies have indicated that hypothermia was a critical factor to hepatic ultrastructural changes, notably autophagy (Salas et al., 1977). In addition, autophagy has been described in many studies that it constitutes a strategy to adapt to and cope with stress by removing misfolded proteins and damaged organelles (Marino et al., 2014; Wu et al., 2019). Recent studies have also shown that rapamycin induced autophagy involved in the immune regulation of *P. vannamei* (Wu et al., 2019). Chlorpyrifos also can induce autophagy that occurs by activating the AMPK/TOR pathway in common carp (Zhang et al., 2019). Accumulating evidence suggests that TOR plays a gating role in autophagy, and its activity is the key to the formation and maturation of autophagy (Wang and Klionsky, 2003). However, the role of miRNA-mediated TOR genes in the occurrence of autophagy has been studied only in invertebrates.

miRNAs, as critical regulators of transcription, RNA processing, RNA stability, and translation, were involved in the regulation of cell proliferation, differentiation, autophagy, and apoptosis regulation (Carthew and Sontheimer, 2009). Many miRNAs have recently been identified to play key roles in regulating autophagy. In the diabetic mesangial cell, Oleanolic acid activation of autophagy via miRNA-142-5p/PTEN signaling attenuates cell injury (Chen et al., 2019). miR-71 and miR-13b are necessary for viral infection and host autophagy of shrimp (He et al., 2017). Furthermore, miRNA regulates autophagy mainly through the regulation of the expression of key proteins in the process of autophagosome formation. ULK1 can be inhibited by mir-20a or mir-106b expression, thereby inhibiting leucine deficiency–induced autophagy (Wu et al., 2012). miR-30a inhibits autophagy by inhibiting the expression of Beclin1 and Atg5 proteins during nucleation and elongation of autophagosomes (Zhai et al., 2013). However, the regulation of autophagosome formation process proteins is more limited. Because the initiation of autophagy is regulated by numerous signaling pathways such as PI3K/AKT/mTOR, ERK1/2, elF-2, AMPK, and MAPK signaling pathway (Lim et al., 2019; Wang et al., 2019), therefore, PI3K/AKT/mTOR signaling pathway is an important upstream regulatory factor on the formation of autophagosomes. For instance, mTOR is a target gene such as miR-7, miR-199a-3p, and miR-100 and can block autophagy induced by the mTOR signaling pathway (Alqurashi et al., 2013). Fortunately, crustacean miRNAs, which are involved in the regulation of autophagy, have also been gradually discovered. Twenty-four miRNAs were also found to play a role in autophagy, phagocytosis, and apoptosis in *Marsupenaeus japonicus* (Tianzhi Huang, 2012). In this context, the posttranscriptional regulation mediated by miRNAs may shed light on the interaction between autophagy and damage.

**FIGURE 9:**
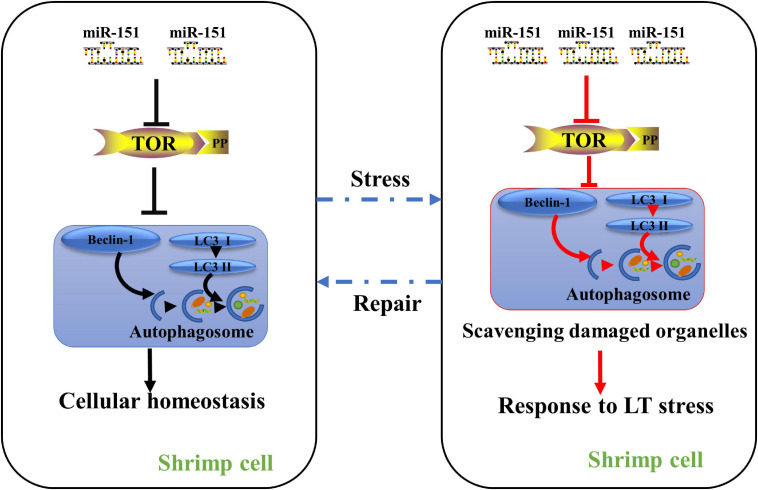
A proposed model for the role of miR-151 in low-temperature tolerance. Under normal physiological conditions, miR-151 mediates the expression of PvTOR to maintain intracellular homeostasis. When the shrimp were exposed to low-temperature stress, inhibiting miR-151 reduces the accumulation of TOR and p-tor, thereby regulating the expression of autophagy-related proteins, resulting in increasing the accumulation of autophagosomes. The accumulated autophagosomes contribute to degrading these organelles such as damaged mitochondria. To alleviate hepatopancreas injury induced by acute low temperature in shrimp. Red lines indicate increased pathways, whereas blank lines indicate maintain intracellular homeostasis pathways.

This data indicate that 36 differentially expressed miRNAs were identified under low-temperature stress, and their target genes were significantly enriched in endocytosis and mTOR signaling pathway. Studies show that mTOR can drive protein synthesis (Aramburu et al., 2014). Shrimps need to consume energy under low-temperature stress. Thus, the expression of pva-miR-151 was decreased at 1.5 h after low-temperature stress, suggesting that down-regulation of pva-miR-151 could partially promote activities of mTOR to protect stressed cells by increasing the rate of biosynthesis and the cellular energy demand. However, constitutively active mTOR could increase ROS production and induced cell damage. Thus, the expression of pva-miR-151 was increased at 3 to 24 h after low-temperature stress, suggesting that up-regulation pva-miR-151 could partially inhibit activities of mTOR activating an autophagic response that clears protein aggregates and damaged organelles and improves cell survival. Additionally, low-temperature stress can induce hepatopancreatic injury and autophagy accumulation. The results revealed that miRNAs are involved in the regulation of autophagy under low-temperature stress. Currently, the mechanism associated with autophagy and miRNA, which controlled shrimp response to low-temperature stress, is still unclear. In this study, we characterized the expression patterns and biological functions of pva-miR-151 in shrimp and provided a new regulatory mechanism by which pva-miR-151 mediated *PvTOR* regulation in shrimp response to low-temperature stress. Sequence analysis revealed that mature pva-miR-151 is completely conserved in mammals, and LUC reporter experiment confirmed that the pva-miR-151 precursor can be folded into a hairpin structure. In addition, pva-miR-151 directly targeted the 3′-UTR gene to regulate *PvTOR* expression by confirming experimentally. As reported, the significant reduction of miRNA-151 was associated with severe liver injury (Cheng et al., 2018). Up-regulation of miR-151-3p was closely related to the overall survival rate in resected cholangiocarcinoma (McNally et al., 2013). The data of this study indicate that the accumulation of autophagy induced by overexpression of pva-miR-151 can alleviate hepatopancreas injury caused by low-temperature stress and reduce the mortality of shrimp and *vice versa* (Figure 9). Autophagy is a necessary process in which cell recycling degrades cytoplasm and organelles by the lysosomal pathway in all eukaryotic cells (Yan et al., 2019). Autophagy has been shown to be a survival mechanism under different stress conditions (Wei et al., 2015; Jing et al., 2018). These findings showed that pva-miR-151 through target gene *PvTOR* regulating the autophagy accumulation involved in *P. vannamei* response.

Loss and damage of circulating hemocytes in shrimp can lead to decreasing the immune ability, augment the susceptibility against pathogens, and even threaten survival, demonstrating that hemocytes play an important role in physiology and immune defense (Smith et al., 1995; Li et al., 2013). It was documented that acute low-temperature stress could result in a reduction of the THC (Qiu et al., 2011). It is suggested that overexpression of pva-mir-151 can alleviate THC reduction caused by low-temperature stress. In crustaceans, there are three morphologically different hemolymph types: hyaline, semigranular, and granular cells (Johansson et al., 2000). Both hyaline and semigranular have phagocytosis. Thus, we speculate that pva-miR-151 might regulate hemolymph autophagy or phagocytosis-related protein expression to inhibit hemolymph apoptosis. There was no evidence in this article to explain this molecular mechanism; the results showed that pva-miR-151 is closely related to shrimp THC, which needs to be investigated in further research.

Taken together, the present study indicated that both pva-miR-151 and *PvTOR* were involved in shrimp response to low-temperature stress. Ectopic expression of pva-miR-151 resulted in low-temperature tolerance phenotype. A low-temperature hypersensitive phenotype was observed in silenced pva-miR-151 in *P. vannamei*. The present study provides a much-needed framework that pva-miR-151 responds to low-temperature tolerance of *P. vannamei* by increased autophagy accumulation and THC under low-temperature stress by its target gene *PvTOR* expression.

## Data Availability Statement

The original contributions presented in the study are included in the article/Supplementary Material, further inquiries can be directed to the corresponding author/s.

## Ethics Statement

The animal study was reviewed and approved by animal protocols conform to the University Animal Care and Use Committee of the South China Normal University.

## Author Contributions

QL: software and writing-original draft preparation. QL, MO, and WD: validation and writing-review and editing. ZL, CL, and FW: prepare the experiment materials. YL and WW: project administration and funding acquisition. All authors have read and agreed to the published version of the manuscript.

## Conflict of Interest

The authors declare that the research was conducted in the absence of any commercial or financial relationships that could be construed as a potential conflict of interest.

## Footnotes

1 https://omictools.com/rnahybrid-tool

2 http://www.targetscan.org/

3 http://www.microrna.org/

4 http://www.geneontology.org/

5 http://www.genome.jp/kegg/

6 http://unafold.rna.albany.edu/?q=mfold

7 https://imagej.nih.gov/ij/

## Supplementary Material

Supplementary Figure 1 The energy dot plot of pva-miR-151 precursor. The hairpin structures of pva-miR-151 precursor were formed by the mfold Web server (http://unafold.rna.albany.edu/?q~=~mfold). The dots represent the superposition of all possible folding within p% of ΔGmfe, the minimum free energy, where *p* is the maximum percent deviation from ΔGmfe. Different colors are used to indicate varying levels of suboptimality. The explanations of relevant parameters were detailed in the mfold Web server.

Supplementary Figure 2 The shrimp were injected with ago-151 or antago-151. After different hours that the efficiency was analyzed by qRT-PCR in hepatopancreas.

Supplementary Table 1 Sequences of PCR primers used in this study.

Supplementary Table 2 Differential miRNA after low-temperature stress 3 h.

Supplementary Table 3 Differential miRNA target genes predicted.

Supplementary Table 4 Differential miRNA target genes by KEGG analysis.

Supplementary Table 5 Sequences of miR-151 (red) and its precursor (blue).
