# Supplementary material for: miR-151 Affects Low-Temperature Tolerance of Penaeus vannamei by Modulating Autophagy Under Low-Temperature Stress
Source: Front Cell Dev Biol. 2021 Apr 9;9:595108. doi: 10.3389/fcell.2021.595108 (PMC8064728; doi:10.3389/fcell.2021.595108)
Supplement: Supplementary Table 1 — Sequences of PCR primers used in this study. [file Table_1.DOCX]

Supplementary Table S1 Sequences of PCR primers used in this study.

| Gene Name | Primer | Gene Name | Primer |
| --- | --- | --- | --- |
| PvTOR-F | GCCAGACATCACGACGCA | PvTOR-R | CCGCCCTGAGCAAACG |
| Pri-151-F | TCCTTCAAGGTAGGCGGTCG | Pri-151-R | GTGAATCTCGCACTTATCTA |
| MiR-151-F | GGACCCCTAGACTGAAGCTC | MiR-151-R | TGGTGTCGTGGAGTCG |
| MiR-151-RT | CTCAACTGGTGTCGTGGAGTCGGCAATTCAGTTGAGCAAACACC | | |
| WT-UTR-F | AATTCTAGGCGATCGCTCGAGGAAGTTCTGTTGGACTATATATATCCACAATAAATTGT | | |
| WT-UTR-R | TTTTATTGCGGCCAGCGGCCGCGCTTCAGTGGGATGGTGT | | |
| MUT-UTR-F | AATTCTAGGCGATCGCTCGAGGCTGCCCTCAACCTTCT | | |
| MUT-UTR-R | TTTTATTGCGGCCAGCGGCCGCATGCCCAACACACAGA | | |
| WT-151-F | CACTGACTGACCCTCAAGGCTTCAGTCTAGCAGGACACAAGGCCTGTTACTAGCAC | | |
| WT-151-R | GCCAAAACCCTCAAGGAGCTTCAGTCTAGCAGCATACAGCCTTCAGCAAGCCTCCA | | |
| MUT-151-F | GTCGAGTGACTGCCTTTGATCATTGTTTC | | |
| MUT-151-R | ACAGTGTAACATTTAAAACTAAAAATATAT | | |
| Pre-151-F | CACTGACTGACACCTCAAAGCTTCAGTCCAGTTGCATACTGAGACAAAAATAGATCTTCCTAAACAGAGAACAGGACACAAGGCCTGTTACTAGCAC  GCCAAAACTTCTCTGTTTAGGAAGATCTATTTTTGTCTCAGTATGCAACTGGACTGAAGCTCTTTGAGGTAGCATACAGCCTTCAGCAAGCCTCCA | | |
| Pre-151-R |  |  |  |
| Pri-151-F | CTCTCAGAGTCAAGAACC | Pri-151-R | GTGAATCTCGCACTTATCTA |
| U6S | CTCGCTTCGGCAGCACA | U6A | AACGCTTCACGAATTTGCGT |
