# Supplementary material for: miR-151 Affects Low-Temperature Tolerance of Penaeus vannamei by Modulating Autophagy Under Low-Temperature Stress
Source: Front Cell Dev Biol. 2021 Apr 9;9:595108. doi: 10.3389/fcell.2021.595108 (PMC8064728; doi:10.3389/fcell.2021.595108)
Supplement: Supplementary Table 5 — Sequences of miR-151 (red) and its precursor (blue). [file Table_5.DOCX]

Supplementary Table S5. Sequences of miR-151 (red) and its precursor (blue).

(A) Primary transcript of miR-151 (pri-pva-miR-151)

CUUUAAUCCUUGAAAUUAGGCAUGUAAUUUCUUUGUCAGAGGUAAGUCAAAAUCGUAUAUGGGUAUAGGUAUUGUUGUUCUCUGUUUAGGAAGAUCUAUUUUUGUCUCAGUAUGCAACUGGACUGAAGCUCUUUGAGGUAUGUAUUCUACAUAUUUUUUGAGAUGUGAUUGCCUGCCAGUUUCUACAAUUUUUGGAUUUUGUAACAUUAUUAUUUUUUUCUAGUUGCAUUUUUUUUUCUUUUGCUUAUAUUGUUCUUCUGAUUUUCUCUACUUGGCCAUUAAUUCAGCUUGAAAGGGAAAAGUGCAAGAAC

(B) Precursor sequence of miR-151 (pre-pva-miR-151)

UUCUCUGUUUAGGAAGAUCUAUUUUUGUCUCAGUAUGCAACUGGACUGAAGCUCUUUGAGGU

(C) Mature sequence of miR-151 (pva-miR-151)

ACUGGACUGAAGCUCUUUGAGG
